# Direct Evidence of Dynamic Metal Support Interactions in Co/TiO_2_ Catalysts by Near-Ambient Pressure X-ray Photoelectron Spectroscopy

**DOI:** 10.3390/nano13192672

**Published:** 2023-09-29

**Authors:** Davide Salusso, Canio Scarfiello, Anna Efimenko, Doan Pham Minh, Philippe Serp, Katerina Soulantica, Spyridon Zafeiratos

**Affiliations:** 1 European Synchrotron Radiation Facility, CS 40220, CEDEX 9, 38043 Grenoble, France; davide.salusso@esrf.fr; 2 Centre RAPSODEE UMR CNRS 5302, IMT Mines Albi, Université de Toulouse, Campus Jarlard, CEDEX 09, 81013 Albi, France; canio2sca@gmail.com (C.S.); doan.phamminh@mines-albi.fr (D.P.M.); 3 Laboratoire de Physique et Chimie des Nano-Objets (LPCNO), Université de Toulouse, INSA, UPS, CNRS, LPCNO, 135 Avenue de Rangueil, 31077 Toulouse, France; ksoulant@insa-toulouse.fr; 4 LCC, CNRS-UPR 8241, ENSIACET, Université de Toulouse, 31030 Toulouse, France; philippe.serp@ensiacet.fr; 5 Interface Design, Helmholtz-Zentrum Berlin für Materialien und Energie GmbH (HZB), Albert-Einstein-Str. 15, 12489 Berlin, Germany; anna.efimenko@helmholtz-berlin.de; 6 Energy Materials In-Situ Laboratory Berlin (EMIL), Helmholtz-Zentrum Berlin für Materialien und Energie GmbH (HZB), Albert-Einstein-Str. 15, 12489 Berlin, Germany; 7 Institut de Chimie et Procédés Pour l’Energie, l’Environnement et la Santé (ICPEES), ECPM, UMR 7515 CNRS—Université de Strasbourg, 25 Rue Becquerel, CEDEX 02, 67087 Strasbourg, France

**Keywords:** SMSI, dynamic surface phenomena, NAP-XPS, NAP-HAXPES, NEXAFS, cobalt nanoparticles, in situ spectroscopy, operando spectroscopy, CO_2_ hydrogenation, tender X-rays

## Abstract

The interaction between metal particles and the oxide support, the so-called metal–support interaction, plays a critical role in the performance of heterogenous catalysts. Probing the dynamic evolution of these interactions under reactive gas atmospheres is crucial to comprehending the structure–performance relationship and eventually designing new catalysts with enhanced properties. Cobalt supported on TiO_2_ (Co/TiO_2_) is an industrially relevant catalyst applied in Fischer−Tropsch synthesis. Although it is widely acknowledged that Co/TiO_2_ is restructured during the reaction process, little is known about the impact of the specific gas phase environment at the material’s surface. The combination of soft and hard X-ray photoemission spectroscopies are used to investigate in situ Co particles supported on pure and NaBH_4_-modified TiO_2_ under H_2_, O_2_, and CO_2_:H_2_ gas atmospheres. The combination of soft and hard X-ray photoemission methods, which allows for simultaneous probing of the chemical composition of surface and subsurface layers, is one of the study’s unique features. It is shown that under H_2_, cobalt particles are encapsulated below a stoichiometric TiO_2_ layer. This arrangement is preserved under CO_2_ hydrogenation conditions (i.e., CO_2_:H_2_), but changes rapidly upon exposure to O_2_. The pretreatment of the TiO_2_ support with NaBH_4_ affects the surface mobility and prevents TiO_2_ spillover onto Co particles.

## 1. Introduction

Oxide-supported metal catalysts play a significant role in a wide range of industrial chemical processes, while they are crucial to the production of sustainable and clean energy from renewable resources [1,2]. The interaction between the metal and the support is critical for the catalytic performance since it controls important characteristics of the catalyst, such as the dispersion, electronic structure, and stability of the active phase [3]. Although the strength of the metal–support interaction is influenced by many different factors, reducible oxides are generally acknowledged to promote stronger interactions with the metal than the non-reducible ones [3,4,5].

Pt supported on TiO_2_ is the archetype of the so-called strong metal–support interaction (SMSI) systems, although other Pt-group metals on TiO_2_ have also been widely investigated [3,4,5]. It is now well established that metal–support interactions are dynamic and their strength may significantly vary depending on the gas environment, ranging from simple charge transfer to more extensive mass transport and surface restructuring [6]. Therefore, a fundamental understanding of SMSI phenomena is of paramount interest for optimizing catalytic performance and designing innovative catalytic systems.

Although the SMSI effect was initially described almost 50 years ago [7], our understanding of it is far from comprehensive, and it remains one of the most researched areas in heterogeneous catalysis [3,4,5,8]. The recent breakthrough developments of advanced material characterization techniques, such as environmental high-resolution transmission electron microscopy (HRTEM), allowed us to visualize the dynamic interplay at the metal–oxide interface during operation, which was not feasible in ex situ and postmortem studies [9,10,11]. HRTEM studies can observe in real time the encapsulation of metal clusters by a thin layer of reduced titanium oxide under reducing conditions, and the formation of a thicker titania overlayer upon subsequent oxidative treatment [9,10,11].

Although less studied than Pt-group metals, transition metals, such as Ni and Co supported on TiO_2_, are known to undergo restructuring processes under reaction conditions [10]. In particular, encapsulation of Co nanoparticles under a thin reduced titania layer has been observed after H_2_ treatment of Co/TiO_2_ catalysts [12,13,14]. Deeper understanding of these interactions is essential in order to improve the performance of such catalysts in industrial applications. For example, Co/TiO_2_ is one of the most industrially applied Fischer−Tropsch synthesis (FTS) catalysts [2,15]. FTS is used to synthesize diesel from biomass feedstocks, among other products. As for Pt/TiO_2_ catalysts, microscopy techniques are primarily used in order to visualize changes in the Co-TiO_2_ interface in response to the gas phase environment [12,13,16]. However, SMSI phenomena over Co/TiO_2_ catalysts have been mostly noticed by ex situ experiments in which the sample was transferred to the microscope after reduction/oxidation gas treatments. In addition, due to the low contrast difference between Ti and Co atoms, in situ HRTEM studies are more challenging than those over Pt/TiO_2_.

Instead, X-ray photoelectron spectroscopy (XPS), due to its chemical specificity and surface sensitivity, is a powerful tool to study chemical and structural transitions of Co/TiO_2_ upon interaction with gas atmospheres. In addition, photoemission experiments are quantitative and give an average information over the entire sampling geometric area (around 100 µm [17]), providing a quite representative image of the overall sample structure. The recent development of XPS spectrometers capable of operating at pressures of a few tens of millibars [18,19,20], referred to as near-ambient pressure XPS (NAP-XPS), provides new opportunities to study SMSI process in reaction environments. However, only a few NAP-XPS studies exist on SMSI effects in TiO_2_-supported catalysts [21,22,23] and to the best of our knowledge, none concerning the Co/TiO_2_ system.

Herein, we present a synchrotron-based near-ambient pressure X-ray photoelectron and absorption study of cobalt catalysts supported on pure and NaBH_4_-modified TiO_2_ [24], in H_2_, CO_2_:H_2_, and O_2_ gas atmospheres. This work is unique in the field of SMSI investigations in that soft and hard X-rays are coupled to extend the analysis depth of the photoemission process by an order of magnitude (from about 2.5 to 25 nm). The results show a dynamic response of the catalysts to the gas phase environment and reveal critical differences between pure and NaBH_4_-modified Co/TiO_2_ catalysts.

## 2. Materials and Methods

### 2.1. Catalysts Preparation

Initially, commercial TiO_2_-P25 (Evonik) was partially reduced using NaBH_4_ as the reducing agent, leading to the incorporation of Na and B promoters. More specifically, known amounts of NaBH_4_ were dissolved in ethanol in a rotavapor flask and then TiO_2_-P25 powder was added to the solution. The resulting mixture was stirred for 1 h (25 °C, 500 mbar) and subsequently, the ethanol was removed using a rotavapor (80 °C, 150 mbar for 30 min and then 75 mbar for 30 min). Next, the sample was dried overnight at 120 °C in a static oven and then treated in a tubular oven under argon at 320–370 °C for 15 min. Afterwards, the resulting blue product was recovered under Ar and then washed three times with distilled water, followed by washing with absolute ethanol. Finally, the product was dried for 15 h at 150 °C under argon. Textural, chemical, and crystallite properties of the TiO_2_ and m-TiO_2_ supports are given in Table S1.

Subsequently, modified and unmodified TiO_2_ were used to prepare 10 wt% Co-based catalysts by conventional incipient wetness impregnation. To do so, the TiO_2_ supports were placed in a Schlenk flask and degassed for 2 h under vacuum at 150 °C (oil bath temperature). After cooling down at room temperature, an aqueous metal precursor solution (Co(NO_3_)_2_·6H_2_O) was added under vacuum and continuous stirring. The resulting mixture was sonicated for 30 min, followed by 30 min of stirring. The sonication/stirring sequence was repeated 4 times. The mixture was dried for 24 h at 80 °C followed by 12 h at 120 °C in a static furnace. Finally, the powder was calcined for 4 h at 460 °C under Ar. Hereafter, the unmodified and modified catalysts will be referred to as Co/TiO_2_ and Co/m-TiO_2_, respectively. The bulk atomic concentration (at%) of the various catalysts’ components, as calculated by inductively coupled plasma optical emission spectroscopy (ICP-OES) measurements, was 9.6 at% Co for Co/TiO_2_ and 8.0 at% Co, 1.4 at% Na, and 0.2 at% B for Co/m-TiO_2_. Because of the low contrast difference between Ti and Co, electron microscopy could not be used to assess the size and distribution of Co particles. Instead, the cobalt crystallite size was determined from XRD measurements using the Scherrer equation. The quantification of the metallic Co crystallite size of the reduced catalysts was not possible due to the overlap of various crystallographic phases. In the case of calcined catalysts, the crystallite size of Co_3_O_4_ was determined to be around 37 nm and 44 nm for Co/TiO_2_ and Co/m-TiO_2_, respectively.

### 2.2. Spectroscopic Measurements

Near-ambient pressure X-ray photoelectron spectroscopy (NAP-XPS) and near-ambient pressure hard X-ray photoelectron spectroscopy (NAP-HAXPES) combined with near edge X-ray absorption fine structure spectroscopy (NEXAFS), were applied to probe the surface state of the pre-reduced Co/TiO_2_ and Co/m-TiO_2_ catalysts. Experiments were performed at the new CAT branch of the dual-colour Energy Materials In-Situ Laboratory (EMIL) beamline (CAT@EMIL) at the synchrotron radiation facility BESSY II of the Helmholtz Zentrum, Berlin [17,25]. A specificity of the CAT@EMIL beamline is the possibility to use soft and hard X-ray radiation (in our case, 4.9 keV, thus in the energy border between tender and hard X-rays) provided by two undulators, UE48 and CPMU17,monochromatized by plane-grating monochromator (PGM) and a double crystal monochromator (DCM), respectively, and focused to the sample position by means of a set of optical mirrors in a single experiment. The variability of the incident photon energy permits non-destructive depth profiling from the extreme surface to the subsurface. The experimental station hosts SPECS PHOIBOS 150 NAP analyzer with a 2D-CMOS detector.

The two catalysts were initially reduced ex situ at 350 °C in 1 bar 40% H_2_/Ar flow for 4 h. The reduced powder was pressed into pellets and mounted on a sapphire sample holder between a stainless-steel back-plate and a lid with 5 mm hole. To guarantee the repeatability of the treatment conditions, the two catalysts were put together, in close proximity, on the sample holder (Figure S1). The sample stage was heated from the rear by an IR laser. Gases were introduced into the analysis chamber via calibrated mass flow controllers (Bronkhorst). The gas phase composition was monitored by a differentially pumped quadrupole mass spectrometer (QMS, Pfeiffer PrismaPro) connected to the sample chamber through a leak valve. The NAP-XPS spectra were measured with a PGM (Bestec GmbH, Berlin, Germany) and a 60 um exit slit. The NAP-HAXPES was measured with a DCM (Bestec GmbH, Berlin, Germany) using Si (111) crystal pair.

The two catalysts were first annealed in front of the spectrometer nozzle for 30 min at 350 °C under 5 mbar H_2_ before being cooled to 200 °C to record the spectra in H_2_. Then, the gas atmosphere was changed to a CO_2_:H_2_ (1:2) mixture with an overall pressure of 2.5 mbar and the temperature was raised to 250 °C where a new set of spectroscopic measurements was recorded. Finally, the CO_2_:H_2_ mixture was replaced by 1 mbar O_2_ and the temperature further rose to 350 °C in order to obtain the characteristic spectra of the oxidized catalysts. The NAP-XPS, NAP-HAXPES, and NEXAFS spectroscopic measurements were carried out consecutively on each sample while maintaining stable gas and temperature conditions.

Quantification of the elements was performed after normalization of the XPS spectra intensities by considering the photon flux and the atomic subshell photoionization cross sections. The photoionization cross sections and the inelastic mean free paths (IMFPs) of photoelectrons were obtained by the SESSA (Simulation of Electron Spectra for Surface Analysis) software (version 2.2) [26]. The incident photon flux was measured based on the sample drain current of a cleaned Au foil. The binding energies (BE) of the photoemission peaks presented here are referred to the Fermi edge cut-off position, measured using the same photon energy with the core-level spectrum measured just before. Unless otherwise stated, the accuracy of BE calibration was estimated to be ±0.1 eV. The XPS spectra were analyzed using CasaXPS software (Casa Software Ltd., version 2.3.25). After linear or Shirley background subtraction, the B 1s, Na 1s, and O 1s spectra were fitted by symmetric Gaussian–Lorentzian line shapes, while constrains on the width and the relative BE position of the fitting components were applied.

The excitation photon energy of the NAP-XPS core-level spectra was selected in such a way that the emitted photoelectrons have comparable kinetic energies (KE). Two sets of NAP-XPS spectra with two different KE (about 180 and 450 eV) were measured, corresponding to two analysis depths (a.d.) of about 2.5 and 5 nm, respectively (the information depth is considered to be 3 times the IMFP) [27]. For all NAP-HAXPES experiments discussed in this work, the photon energy was fixed to 4900 eV (information depth about 25  nm).

The Co L_3,2_- and Ti L_3,2_-edge NEXAFS spectra were measured in the total electron yield (TEY) mode, using a Faraday cup installed in the first aperture of the analyzer electrostatic lenses. In general, the analysis depth of NEXAFS measurements in the TEY mode ranged between 5 and 10 nm [28].

## 3. Results and Discussion

### 3.1. Chemical State Measured in H_2_ and CO_2_:H_2_ Gas Atmospheres

**Figure 1:**
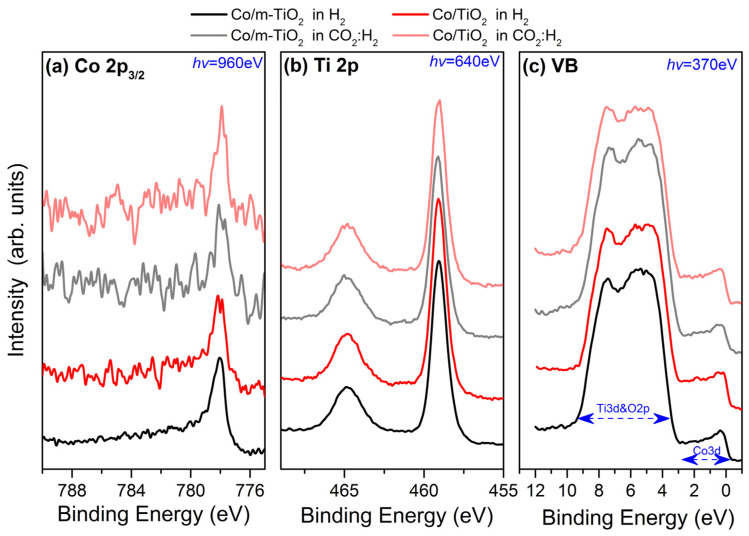
(**a**) Co 2p_3/2_, (**b**) Ti 2p, and (**c**) valence band (VB) NAP-XPS spectra of Co/m-TiO_2_ (black lines) and Co/TiO_2_ (red lines) catalysts measured under 5 mbar H_2_ at 200 °C (dark-colored lines) and 2.5 mbar CO_2_:H_2_ at 350 °C (light-colored lines). Spectra are normalized to the same height to facilitate peak shape comparison. The excitation photon energies for each spectrum (in blue) are selected so as to give photoelectrons with kinetic energies around 180 eV. The estimated analysis depth of the presented spectra is about 2.5 nm (calculated as 3 times the inelastic mean free path of the photoelectrons).

Figure 1 shows the Co and Ti 2p core level and valence band spectra of the two catalysts measured at 200 °C in 5 mbar H_2_ and at 250 °C in a 2.5 mbar CO_2_:H_2_ mixture. The peak shape and the binding energy of the Co 2p_3/2_ peak at 778 ± 0.1 eV (Figure 1a) is characteristic of metallic Co [29,30]. The two catalysts have almost identical Co 2p_3/2_ spectra, suggesting that the modification of TiO_2_ support by NaBH_4_ does not notably affect the cobalt chemical state. In addition, Co 2p_3/2_ seems unaffected after switching the gas atmosphere to the CO_2_:H_2_ mixture, as validated by the NEXAFS data (vide infra). The Ti 2p spectra (Figure 1b) are composed of two peaks at 459.1 eV and 464.8 eV, which are ascribed to Ti 2p_3/2_ and Ti 2p_1/2_ in TiO_2_, respectively. The Ti 2p peaks of both samples are identical in H_2_ and CO_2_:H_2_ atmospheres and correspond to the Ti^4+^ ions of stoichiometric TiO_2_ [22]. It is interesting that there are no evident peak features at the low BE side of the Ti 2p that could be ascribed to Ti^3+^ formation [22]. This shows that TiO_2_ is not reduced under the present hydrogen treatment, a result which is consistent with our NEXAFS Ti L-edge spectra (vide infra).

The valence band (VB) is dominated by the Ti 3d and O 2p states between 10 eV and 3 eV [31] and the characteristic Co 3d sharp cut-off at the Fermi level [32]. The relative height of the two distinct features at 5.2 and 7.2 eV is sensitive to the type of TiO_2_ polymorph (a-TiO_2_ (anatase) or r-TiO_2_ (rutile)) [31]. The VB spectra of the Co/TiO_2_ and Co/m-TiO_2_ catalysts look alike, confirming the core-level spectra, which indicated that the two catalysts have identical Co and Ti oxidation states. In addition to that, the stability of the two features corresponding to Ti 3d and O 2p states indicates that there is no phase transition of TiO_2_ under the reaction conditions employed.

**Figure 2:**
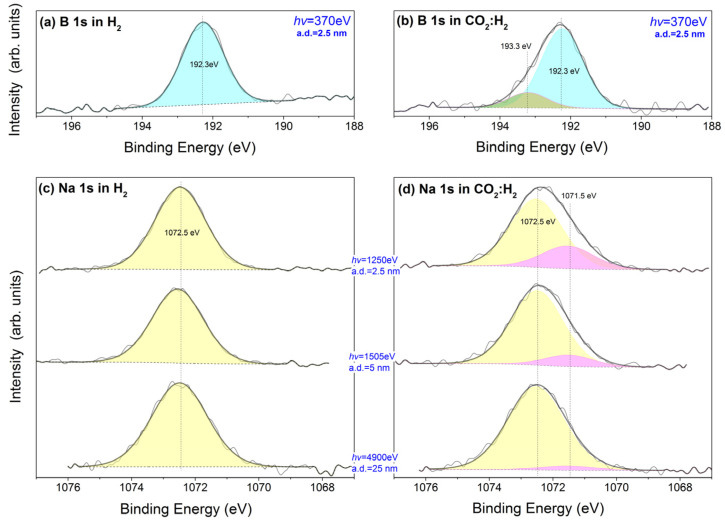
B 1s core level spectra of Co/m-TiO_2_ catalyst measured under (**a**) 5 mbar H_2_ at 200 °C and (**b**) 2.5 mbar CO_2_:H_2_ at 350 °C. Na 1s spectra of Co/m-TiO_2_ catalyst under (**c**) 5 mbar H_2_ at 200 °C and (**d**) 2.5 mbar CO_2_:H_2_ at 350 °C. Spectra are normalized to the same height to facilitate peak shape comparison. The Na 1s spectra were recorded at 3 different excitation photon energies corresponding to 3 different analysis depths (a.d.) (indicated in blue).

The presence of B 1s and Na 1s photoemission peaks (Figure 2) indicates that Na and B remain on Co/m-TiO_2_ sample surface after the NaBH_4_ treatment. Previous reports have shown that the BE of the B 1s is sensitive to the boron local chemical environment [33,34,35,36]. In particular, the B 1s peak of anionic B^2−^ (i.e., TiB_2_ or CoB) and cationic B^3+^ (i.e., B_2_O_3_) appears around 187.5 eV and 193 eV, respectively, while B occupying substitutional or interstitial TiO_2_ sites is found between 190 and 192 eV [33,34,35,36]. Under H_2_, the BE of B 1s peak was measured at 192.3 ± 0.1 eV (Figure 2a), which suggests that B most probably forms an oxide, rather than being integrated in the TiO_2_ structure by substitution of TiO_2_ sites. Moreover, the BE at 192.3 eV is significantly lower than that reported for the common B_2_O_3_ oxide, indicating that boron is partially reduced forming a substoichiometric oxide (e.g., boron suboxide, B_6_O). The low width (1.35 eV) and the symmetry of the peak shape under H_2_ is a sign that this is the unique boron oxidation state. Under CO_2_:H_2_, the B 1s peak becomes broader and shows an asymmetry at the high BE side (Figure 2b). Curve fitting of the B 1s suggests the presence of an additional B 1s peak at 193.3 eV, which is compatible with oxidation of B-suboxide to B_2_O_3_. This indicates the affinity of B towards the CO_2_ present in the reaction mixture, which is considered a mild oxidant. One should note here that the QMS results showed negligible H_2_O production under the low-pressure conditions employed suggesting that CO_2_ is the only oxidant present in the gas phase. 

The Na 1s spectra in H_2_ (Figure 2c) show a single symmetric peak at 1072.5 eV, which is characteristic of Na_2_O and/or ionic Na species bound to the surrounding support through –O–Na linkages [37,38]. For the sake of convenience, the two species mentioned above are abbreviated as NaO_x_. The formation of sodium carbonate or hydroxide species is unlikely, since in that case, the Na 1s would appear at a considerably lower BE [39]. When the catalyst is exposed to CO_2_:H_2_ (Figure 2d), an evident shoulder appears at the low BE side of the Na 1s peak. The Na 1s curve fitting shows the presence of an additional Na 1s component shifted by 1 eV towards a lower BE (1071.5 eV). This peak has been previously assigned to sodium titanate species [40,41]. The depth-dependent Na 1s spectra included in Figure 2d clearly show surface enrichment of the 1071.5 eV peak as compared to the one at 1072.5 eV. This signifies that sodium titanates are formed on top of NaO_x_ species.

**Figure 3:**
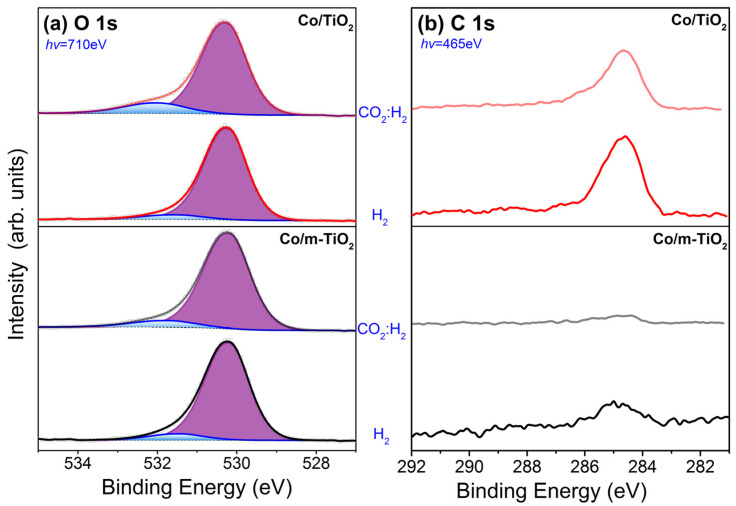
(**a**) O 1s and (**b**) C 1s spectra of Co/TiO_2_ and Co/m-TiO_2_ catalyst measured under 5 mbar H_2_ at 200 °C and 2.5 mbar CO_2_:H_2_ at 350 °C. The O 1s spectra are normalized to the same height while those of C 1s are not.

The O 1s and C 1s spectra are presented in Figure 3. The peak at 530.3 ± 0.1 eV, due to lattice TiO_2_ species [42], dominates the O 1s spectra of the two catalysts (Figure 3a). No discernible differences can be observed in the O 1s peaks of the two catalysts, thereby hindering the ability to differentiate between the contributions of B and Na oxides in the overall peak. Nevertheless, curve fitting of the O 1s peak allows us to distinguish a small O 1s component at 531.6 ± 0.2 eV, typically connected to surface C=O and/or surface adsorbed −OH groups [42]. Since the contribution of C=O is minor in the C 1s peak (vide infra), the component at 531.6 eV should be mainly attributed to −OH species. This is confirmed by depth-dependent O 1s data (Figure S2), which demonstrate a decrease in the 531.6 eV component at deeper analysis depths. In the case of the Co/TiO_2_ sample, the fraction of the OH-peak increases and shifts to higher BEs in CO_2_:H_2_ suggesting a higher abundance of oxygenated species in this case.

The C 1s peak at 284.6 ± 0.1 eV (Figure 3b) is due to C-C and C-H carbon bonds of adventitious hydrocarbons, as could be anticipated for samples that have previously been exposed to air. Notably, the amount of carbon species is more important for Co/TiO_2_ than Co/m-TiO_2_ suggesting that the NaBH_4_ treatment influences the reactivity of TiO_2_ towards carbon. The C 1s spectra in H_2_ and the CO_2_:H_2_ mixture appear very similar, apart from a decrease in the peak intensity under reaction conditions, which is translated to less adsorbed carbon. A small feature at the high-BE side of the C 1s peak in CO_2_:H_2_ can be attributed to the formation of C-O and/or hydroxy species. However, the absence of a peak at around 290 eV can safely exclude the formation of carbonates [43].

**Figure 4:**
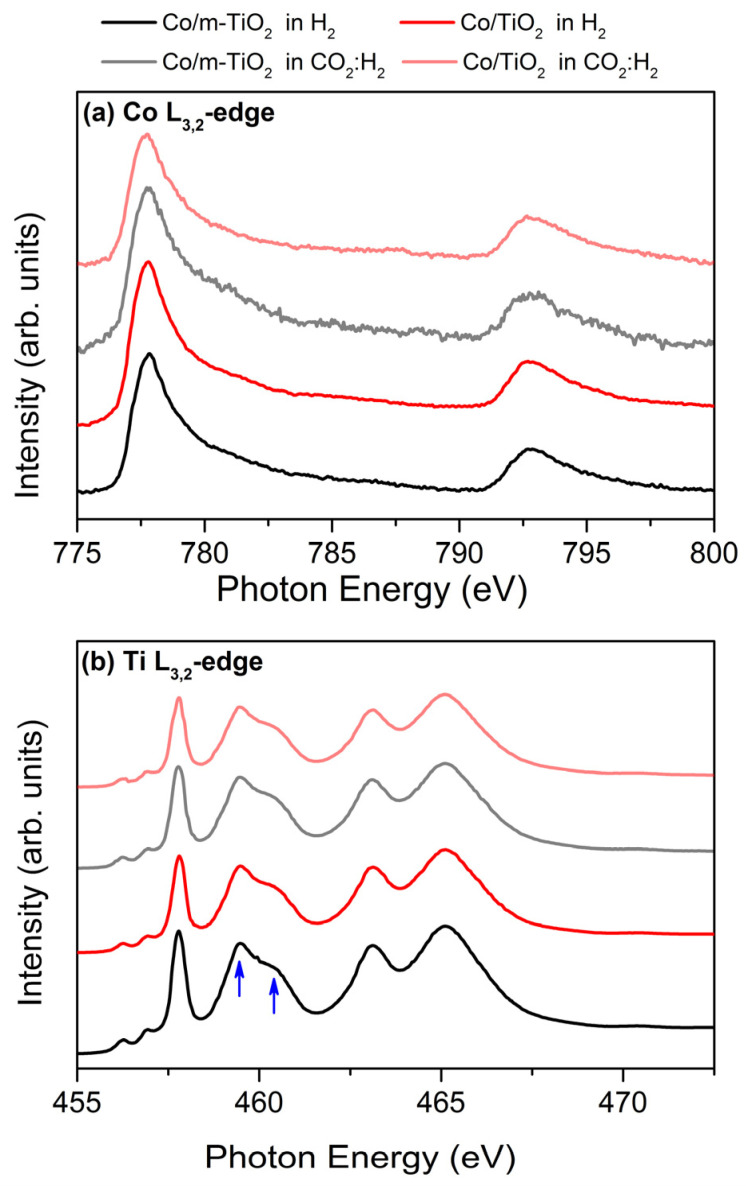
Normalized (**a**) Co L_3_,_2_-edge and (**b**) Ti L_3_,_2_-edge NEXAFS spectra of Co/m-TiO_2_ (black lines) and Co/TiO_2_ (red lines) catalysts measured under 5 mbar H_2_ at 200 °C (dark-colored lines) and 2.5 mbar CO_2_:H_2_ at 350 °C (light-colored lines).

The Co and Ti L-edge X-ray absorption spectra (Figure 4) provide fine details about the electronic and geometric structure of Co and Ti. The Co L-edge (Figure 4a) is composed of two peaks (i.e., Co L_3_- and L_2_-edges) due to the spin–orbit coupling of the Co 2p states. The sharp rising edge of Co L_3_ with the intense maxima at 778.7 eV, as well as the absence of fine structure features at the high photon energy side of the peak, are typical characteristics of metallic Co states [22,30]. Notably, the Co L-edge of the two catalysts is similar and appears to be unaffected by the gas phase conditions, confirming the NAP-XPS results.

The Ti L-edge spectra are included in Figure 4b. The edge splits into two peaks due to the spin–orbit coupling of the Ti 2p states, similar to the Co L-edge. Note that the peak intensity ratio between the two peaks around 460 eV (indicated by the arrows in the figure) is sensitive to the TiO_2_ crystal symmetry. In particular, the lower photon energy spectral feature is considerably greater in height for a-TiO_2_ compared to r-TiO_2_ [22,44]. The Ti L-edge absorption profiles in Figure 4b, including the peak features around 460 eV, correspond to previously reported spectra of a-TiO_2_ [22,44]. Notably, all the Ti L-edges are comparable in terms of spectral line shape and peak excitation energies, suggesting that the valence state and phase of Ti are not affected by the gas atmosphere, which is consistent with the NAP-XPS results (Figure 1). Please note that, while a-TiO_2_ is obviously the dominant surface state based on VB photoemission and Ti L-edge absorption spectra, the existence of minor quantities of r-TiO_2_ cannot be ruled out since their spectroscopic features are obscured by the strong signal of the dominant a-TiO_2_ phase.

In summary, the analysis of the spectroscopic results reveals that Co stays metallic and TiO_2_ is completely oxidized under the employed H_2_ and CO_2_:H_2_ conditions. The comparability of spectroscopic data from Co/TiO_2_ and Co/m-TiO_2_ samples indicates that modification of TiO_2_ by Na and B does not influence the surface chemical state or the electronic structure of Co/TiO_2_ catalyst. Although the CO_2_:H_2_ mixture induces further boron oxidization and promotes sodium titanate formation compared to their state in H_2_, it has no effect on Co or TiO_2_. These findings are more consistent with a static surface configuration than the well-documented dynamic evolution of SMSI systems. This can be justified by the chemical potential of the gas phase, which is highly reducing in both H_2_ and CO_2_:H_2_ atmospheres. Therefore, below, we investigate the surface dynamics under oxidative conditions by changing the gas environment to O_2_.

### 3.2. Surface Transformation upon Exposure to O_2_

**Figure 5:**
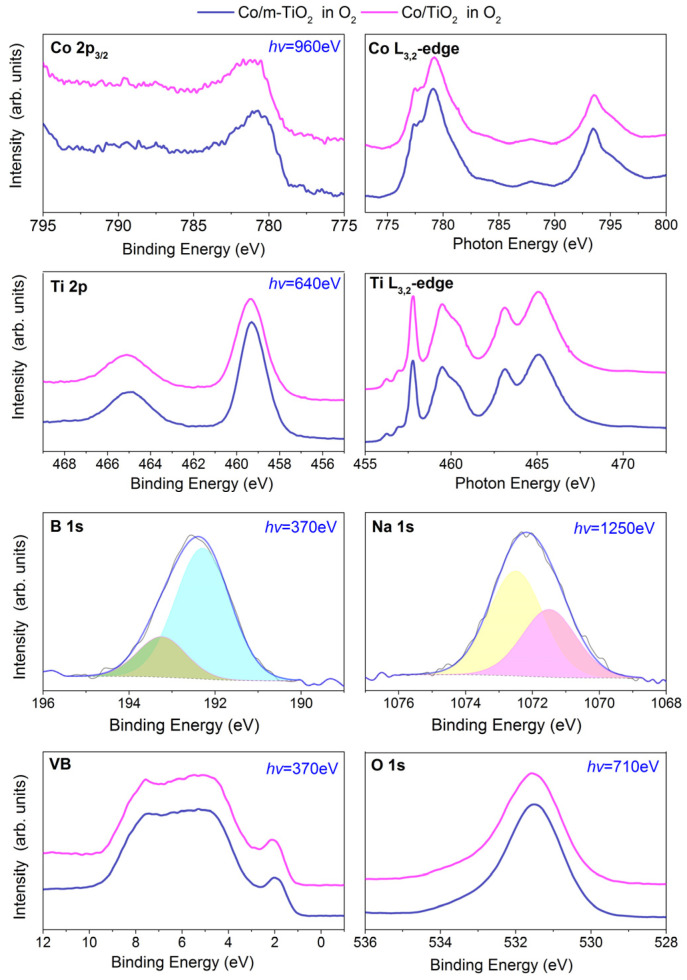
Co 2p_3/2_, Ti 2p, B 1s, Na 1s, valence band (VB) NAP-XPS, and Co and Ti L_3,2_-edge spectra of Co/m-TiO_2_ (blue lines) and Co/TiO_2_ (magenta lines) catalysts measured in 1 mbar O_2_ at 350 °C. The excitation photon energies for each NAP-XPS spectrum (shown in blue) are selected so as to produce photoelectrons with kinetic energies around 180 eV. The estimated information depth is about 2.5 nm (calculated as 3 times the inelastic mean free path of the photoelectrons). The presented spectra are normalized to the same height.

The surface transformation of the reduced samples in an O_2_ atmosphere is examined next. Figure 5 shows the NAP-XPS and NEXAFS spectra of Co/m-TiO_2_ and Co/TiO_2_ recorded in 1 mbar O_2_ at 350 °C. The Co 2p photoemission peaks (Figure 5) become broader and shift to higher BEs in O_2_ compared to the previously recorded spectra in CO_2_:H_2_. This, together with the presence of the low intensity satellite feature around 790 eV, suggests complete oxidation to Co_3_O_4_ [22,29,30]. This is supported by the NEXAFS Co L-edge in Figure 5 (top, right), which is typical of a bulk Co_3_O_4_ spinel structure [22,29,30]. The Ti 2p and Ti L-edge spectra look identical to those recorded in CO_2_:H_2_, which indicates that the a-TiO_2_ phase is preserved in O_2_. The analysis of the B 1s and Na 1s peaks reveals that the proportion of the components at 193.3 eV and 1071.5 eV is enhanced in O_2_ compared to CO_2_:H_2_. (see Figure 2). This supports the hypothesis that the presence of oxidants in the gas environment, including mild oxidants like CO_2_ (Figure 2), promotes the synthesis of Na-titanate and B_2_O_3_.

The spectra of the valence band region confirm the interpretation of the core level peaks. More specifically, the distinct feature appearing at around 2 eV is due to Co 3d states [45], which upon cobalt oxidation are shifted 2 eV below the Fermi edge. On the contrary the features at 10 eV–3 eV region, corresponding primarily to Ti 3d-O 2p states, are identical to those found in H_2_ and H_2_:CO_2_ (Figure 1) confirming the NEXAFS findings. The O 1s spectra of the two samples become significantly broader in O_2_ compared to the previous reduced state (the FWHM of the O 1s peak increases from around 1.3 to 1.8 eV) and shifts by about 0.2 eV to lower BEs. These changes can be understood by the appearance of a new O 1s component due to Co_3_O_4_; however, the absence of clear spectral features in the O 1s peak makes O 1s curve fitting ambiguous, and therefore will not be attempted in this case.

### 3.3. Effect of the Gas Atmosphere on the Surface Composition

**Figure 6:**
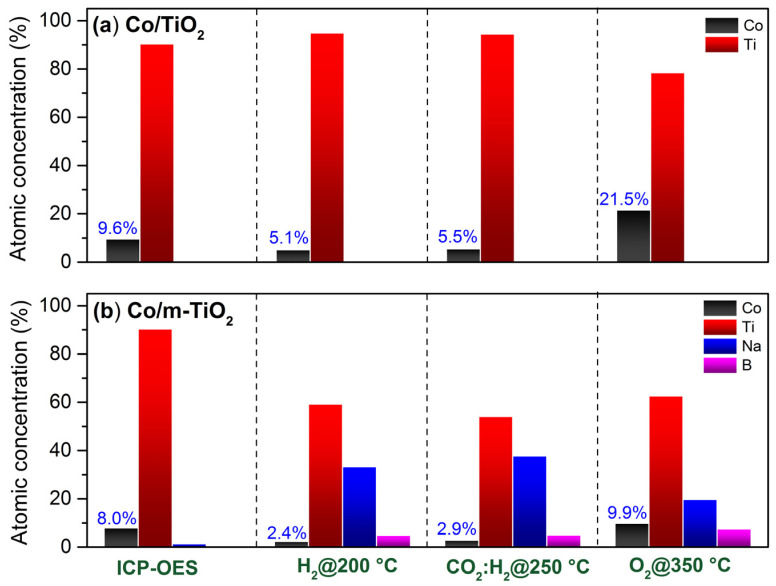
Comparison of atomic concentrations determined from NAP-XPS spectra (analysis depth 2.5 nm) acquired in H_2_, CO_2_:H_2_, and O_2_ atmospheres at the temperatures indicated at the bottom of the graph for (**a**) Co/TiO_2_ and (**b**) Co/ TiO_2_ catalysts. For comparison, the bulk atomic concentrations from ICP-OES measurements are presented. Above the black bars, the atomic concentration of cobalt is indicated.

Due to its high surface sensitivity, photoemission spectroscopy is a powerful tool to quantify the atomic concentration at the very surface. Figure 6 compares the surface atomic composition of the Co/m-TiO_2_ and Co/TiO_2_ catalysts in reducing, reaction, and oxidizing gas environments calculated from the NAP-XPS data. The bulk concentration based on the ICP-OES analysis is included for comparison. In the NAP-XPS calculations, it is assumed that the sample is homogeneous over the analyzed depth. Noteworthily, for layered samples (as will be discussed later) this approximation overestimates the concentration from the top layer relative to that of the layers below. Therefore, one should be cautious when comparing the absolute values of the NAP-XPS and ICP-OES results. Nevertheless, the quantitative NAP-XPS analysis can be used for comparisons to reveal modifications of the surface composition as a function of the gas environment.

In H_2_ and CO_2_:H_2_ atmospheres, the Co surface concentration of Co/TiO_2_ (Figure 6a) is about half of that found by ICP-OES, indicating that the surface is enriched with TiO_2_. However, when the gas environment is changed to O_2_, the Co concentration increases significantly, greatly beyond that of ICP-OES. This reflects a dynamic response of the surface composition to the gas atmosphere, with TiO_2_ segregating over Co in reducing conditions, and Co re-emerging back on the surface in an oxidizing atmosphere.

Significant differences between bulk and surface compositions were also found for Co/m-TiO_2_ (Figure 6b). In particular, in H_2_ and CO_2_:H_2_, the surface contains about 3 times less Co and almost 25 times more Na and B compared to the bulk concentration of these elements. This is a sound evidence that Na and B are the dominant elements on the sample surface. The surface concentration of Co, Ti, and B increase in O_2_ compared to the previous state in CO_2_:H_2_, at the expense of Na. As shown in Figure 5, this is accompanied by enhancement of the component related to Na-titanate species. Thus, when exposed to oxidizing conditions, a portion of the TiO_2_ support interacts with NaO_x_ at the surface to create Na-titanates. This is not necessarily a solid-state reaction, but might simply be due to sodium migration within the TiO_2_ lattice. As was showed earlier, Na^+^ ions may be introduced into the TiO_2_ host without damaging the anatase structure or altering the TiO_2_ oxidation state [46].

### 3.4. Depth Distribution of the Catalyst Components

By taking advantage of the tunability of synchrotron radiation, one can vary the kinetic energy of photoelectrons and in turn the information depth of the measurements. This approach, usually referred to as depth-profiling, allows the quantification of the composition at various depths, which is particularly useful in cases of layered surface morphologies as is obviously the case here. The combination of NAP-XPS and NAP-HAXPES enables the excitation photon energy to be varied between 370 and 4900 eV, thereby extending the sample depth from 2.5 to 25 nm. More details about the relationship between photon energy and analysis depth can be found in the Supplementary Material.

**Figure 7:**
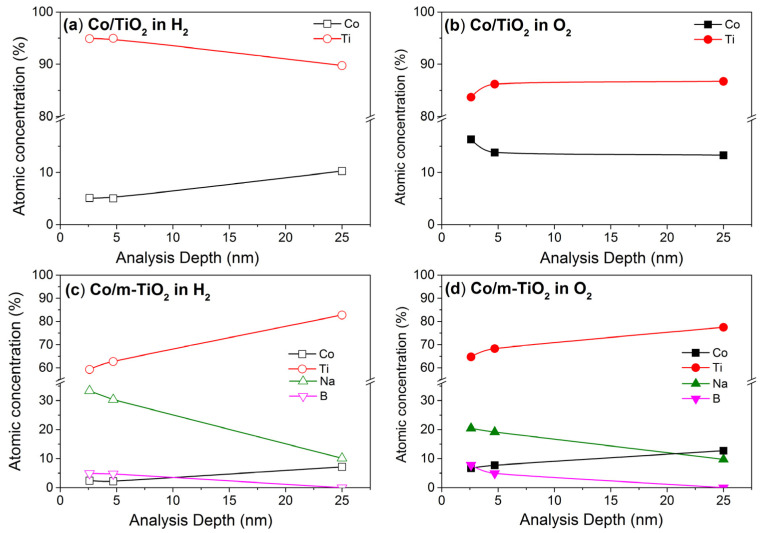
The atomic concentration calculated based on NAP-XPS and NAP-HAXPES spectra as a function of the analysis depth (defined as 3 times the IMFP) measured at 200 °C in 5 mbar H_2_ (**a**,**c**) and (**b**,**d**) at 350 °C in 1 mbar O_2_ for Co/TiO_2_ (**a**,**b**) and (**c**,**d**) Co/m-TiO_2_ catalysts.

**Figure 8:**
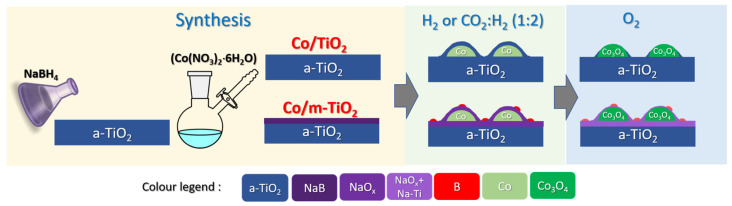
Illustration of the proposed surface arrangement of Co/TiO_2_ and Co/m-TiO_2_ catalysts in H_2_ and CO_2_:H_2_ atmospheres as well as in subsequent exposure to O_2_.

Figure 7a shows the evolution of Co and Ti %at for Co/TiO_2_, as a function of the analysis depth measured at 200 °C in 5 mbar H_2_. The Ti atomic fraction is clearly enhanced at the surface (up to 5 nm) compared to the subsurface measurement (25 nm), which can be associated with the encapsulation of Co under a thin TiO_2_ overlayer (shown in the graphical illustration in Figure 8). This finding is consistent with the low surface fraction of Co compared to the bulk concentration shown in Figure 6a. This is a classical manifestation of SMSIs between Co and TiO_2_, where the support, in a partially reduced state, migrates onto Co particles during the reduction process [12]. Despite the fact that the SMSIs are frequently accompanied by partially reduced TiO_2_ species (e.g., Ti^3+^) [12,22], in our case, the Ti 2p and Ti L-edge spectra did not show any evidence of reduced TiO_2_ for all analysis depths. This implies that this configuration existed prior to the NAP-XPS studies, most likely happening during the ex situ reduction pretreatment at 1 bar (see Section 2.1), which is much more aggressive than the reducing conditions in the NAP-XPS chamber. Under the CO_2_:H_2_ reaction conditions, the depth profile measurements are qualitatively similar to those presented above under H_2_, suggesting no significant surface reconstruction during reaction, in agreement with the findings of Figure 6a.

In the O_2_ atmosphere (Figure 7b), the trend between the two elements is reversed compared to H_2_, with the Co fraction strongly increased near the surface (2.5 nm) but stabilized when deeper layers are probed. This is a clear indication that the thin TiO_2_ overlayer on Co, which manifested in H_2_ and CO_2_:H_2_ atmospheres, retreats under oxidative conditions and cobalt is exposed on the surface (Figure 8). The reversibility of the TiO_2_ spillover effect, where Co is oxidized and segregates above TiO_2_, has been suggested in early SMSI literature [9]. However, to our knowledge this is one of the rare cases where the reversibility of the SMSI effect is evidenced in situ with a method other than microscopy.

Figure 7c shows the depth distribution of Co, Ti, Na, and B for the Co/m-TiO_2_ sample under H_2_. The enhanced concentration of Na and B at the more surface-sensitive measurements (i.e., 2.5 and 5 nm) implies that these elements are not equally distributed within the catalyst volume, but preferentially situated on its surface, above Ti and Co (Figure 8). This interpretation agrees with the high surface concentration found for Na and B as compared to the bulk concentration, as shown in Figure 6b.

When the Co/m-TiO_2_ sample is annealed in O_2_ (Figure 7d), the Co and Ti evolution with analysis depth remains qualitatively similar to that observed in H_2_, meaning that B and Na are still dominating the extreme surface. However, a comparison of the Co %at between H_2_ and O_2_ atmospheres reveals that there is more Co under O_2_ for all analysis depths, comparable to the finding for Co/TiO_2_. The rise in Co percentage under O_2_ should be linked to the decrease in Na %at. This may be caused by either a drop in the thickness of the Na layer over the Co_3_O_4_ particles or its full disappearance. The latter scenario is rather improbable due to the fact that depth profiling measurements (Figure 7d) clearly show that it is Na %at which is enhanced at the outer surface compared to the subsurface, and not Co. One can assume that boron oxides are also preferentially located at the extreme surface, since B %at has a similar evolution with Na as a function of the analysis depth (Figure 7d). Therefore, as shown in Figure 8, we propose that the Na and B surface overlayer formed under reducing conditions is maintained under O_2_ but decreases in thickness. One should mention here that depth profile measurements cannot distinguish if the overlayer above cobalt is dense or has a certain porosity that would allow access to gases during reaction. The two morphologies (i.e., porous or dense Na and B surface overlayer) are expected to lead to radical differences in the catalytic performance. In particular, a dense surface layer will block the access of the reactants to cobalt, thus decrease the number of active sites and consequently the catalytic activity. On the contrary, in case of a porous overlayer, not only cobalt sites remain accessible to the reactants, but the additives can also act as promoters, enhancing the catalytic turnover.

The aforementioned analysis suggests that the modification of the TiO_2_ support by Na and B does not affect the chemical state of the cobalt catalyst, but plays a significant role in the arrangement between Co and TiO_2_ by preventing TiO_2_ migration over cobalt. At this stage, it is not clear if the lower surface mobility on m-TiO_2_ is due to the lower reducibility of this support. Under the reduction conditions employed in the NAP-XPS experiment, TiO_2_ was quite stable for both samples. In addition, H_2_-TPR measurements were not conclusive about the effect of Na and B in the reducibility of the TiO_2_. In any case, XPS is the method of choice here since H_2_-TPR lacks the sensitivity to detect surface reduction of TiO_2_, which is critical to identifying SMSIs.

Overall, the goal of this study was to examine the evolution of Co/TiO_2_ catalysts in reactive gas atmospheres while also contributing to our understanding of metal–support interaction mechanisms. The observed surface restructuring accompanied by changes in cobalt phase, seen while switching between reducing and oxidative gas atmospheres, are likely to alter the catalyst’s reactivity. According to our findings, a thin TiO_2_ layer is anticipated to develop on top of the cobalt during H_2_ activation of calcined catalysts. This layer will affect the cobalt particle growth and the accessibility of the reactants over cobalt sites. However, whether the geometric and electronic structure of pre-reduced Co/TiO_2_ catalysts is maintained throughout reaction conditions, or the catalyst undergoes additional alterations, remains open. The measurements in the CO_2_-FTS-relevant conditions (i.e., CO_2_:H_2_ mixture at 250 °C) presented in this paper, suggest that the cobalt chemical state and atomic concentration remain rather stable. In contrast, we found a noticeable change in the chemical state of B and Na in CO_2_-FTS conditions for Co supported on NaBH_4_-modified TiO_2_. In particular, the presence of CO_2_ in the gas phase oxidizes boron and triggers an interaction at the Na-TiO_2_ interface. This trend implies that under realistic high-pressure FTS reaction conditions, B_2_O_3_ and Na-titanates may be the dominating surface species over the Co/m-TiO_2_ catalyst. Furthermore, our findings imply that when cobalt is oxidized, for example by H_2_O production under FTS conditions, unmodified catalysts experience considerable surface restructuring, which is not the case with Co/m-TiO_2_. Although these findings provide important insights into dynamic surface modifications, it is impossible to predict how the catalytic performance will be modified based solely on these data. Before attempting to connect surface states with catalytic performance, further information about the active phase features, such as nanoparticle size, shape, and exposed facets, is necessary.

## 4. Conclusions

In summary, in this work, we combined NAP-XPS, NAP-HAXPES, and NEXAFS spectroscopies to explore the in situ interaction of two Co/TiO_2_ catalysts with H_2_, CO_2_:H_2_, and O_2_ atmospheres. We found that cobalt is reduced to the metallic state under the employed H_2_ and CO_2_:H_2_ conditions, while it is readily transformed to spinel Co_3_O_4_ upon switching to O_2_. On the contrary, the titania chemical state and structure are not affected by the gas treatment with the a-TiO_2_ phase dominating the surface. The treatment of TiO_2_ with NaBH_4_ left significant amounts of Na and B residuals on the surface even after Co deposition in the aqueous metal precursor solution. The restructuring of the Co-TiO_2_ interface under operating conditions was directly observed by quantitative analysis of photoemission results combined with depth-profiling measurements. It was shown that, under reduction conditions, cobalt particles are encapsulated below a stoichiometric TiO_2_ layer. This arrangement seems to be preserved under the employed CO_2_ hydrogenation conditions, but rapidly changes upon exposure to O_2_. The pretreatment of the TiO_2_ support with NaBH_4_ affects the surface mobility of TiO_2_ and, to a large extent, prevents spillover onto cobalt. However, in this case, cobalt is covered under a Na and B layer, probably formed upon reduction. To a large extent, this layer is preserved upon O_2_ exposure, despite a notable decrease in its thickness. Our findings highlight the dynamic behavior of the Co-TiO_2_ interface in reducing and oxidizing gas atmospheres, which is of particular interest to understand the performance of these materials in catalytic applications.

## Data Availability

Data are available on request.

## Supplementary Materials

The following supporting information can be downloaded at: https://www.mdpi.com/article/10.3390/nano13192672/s1, Table S1: Textural, chemical, and crystallite properties of TiO_2_ supports; Figure S1: Photograph of the sample holder and details about the relationship between photon energy and analysis depth. Figure S2: The O 1s spectra of Co/TiO_2_ catalysts measured in 2.5 mbar CO_2_:H_2_ at 350 °C with 3 different excitation photon energies corresponding to 3 different analysis depths (a.d) (indicated in blue). Spectra are normalized to the same height to facilitate peak shape comparison. Refs. [47,48] are cited in Supplementary Materials.
