# Supplementary material for: Direct Evidence of Dynamic Metal Support Interactions in Co/TiO2 Catalysts by Near-Ambient Pressure X-ray Photoelectron Spectroscopy
Source: Nanomaterials (Basel). 2023 Sep 29;13(19):2672. doi: 10.3390/nano13192672 (PMC10574330; doi:10.3390/nano13192672)
Supplement: Supplementary file 1 [file nanomaterials-13-02672-s001.zip › nanomaterials-2626868-supplementary.pdf]

Direct Evidence of Dynamic Metal Support Interactions in Co/TiO2 Catalysts by Near-Ambient Pressure X-ray Photoelectron Spectroscopy

Davide Salusso [1], Canio Scarfiello [2,3,4], Anna Efimenko [5,6], Doan Pham Minh [2], Philippe Serp [4], Katerina Soulantica [3] and Spyridon Zafeiratos [7,*]

[1] European Synchrotron Radiation Facility, CS 40220, CEDEX 9, 38043 Grenoble, France; davide.salusso@esrf.fr
[2] Centre RAPSODEE UMR CNRS 5302, IMT Mines Albi, Université de Toulouse, Campus Jarlard, CEDEX 09, 81013 Albi, France; canio2sca@gmail.com (C.S.); doan.phamminh@mines-albi.fr (D.P.M.)
[3] Laboratoire de Physique et Chimie des Nano-objets (LPCNO), Université de Toulouse, INSA, UPS, CNRS, LPCNO, 135 Avenue de Rangueil, 31077 Toulouse, France; ksoulant@insa-toulouse.fr
[4] LCC, CNRS-UPR 8241, ENSIACET, Université de Toulouse, 31030 Toulouse, France; philippe.serp@ensiacet.fr
[5] Interface Design, Helmholtz-Zentrum Berlin für Materialien und Energie GmbH (HZB), Albert-Einstein-Str. 15, 12489 Berlin, Germany; anna.efimenko@helmholtz-berlin.de
[6] Energy Materials In-Situ Laboratory Berlin (EMIL), Helmholtz-Zentrum Berlin für Materialien und Energie GmbH (HZB), Albert-Einstein-Str. 15, 12489 Berlin, Germany
[7] Institut de Chimie et Procédés Pour l'Energie, l'Environnement et la Santé (ICPEES), ECPM, UMR 7515 CNRS—Université de Strasbourg, 25 Rue Becquerel, CEDEX 02, 67087 Strasbourg, France
* Correspondence: spiros.zafeiratos@unistra.fr

Table S1. Textural, chemical and crystallite properties of the TiO_2 and m-TiO_2 supports

TiO_2-P25

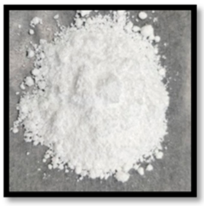

m-TiO_2

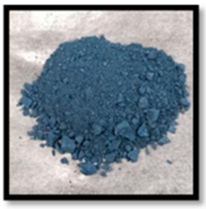

Support	BET surface area ($m^2 \cdot g^{-1}$)	ICP-OES (%wt)		Crystallite size[a)] (nm)		% r-TiO_2[a)]
		Na	B	a-TiO_2	r-TiO_2	
TiO_2	59	-	-	25	44	14.4
m-TiO_2	60	0.52	0.02	25	37	10.6

a) From XRD.

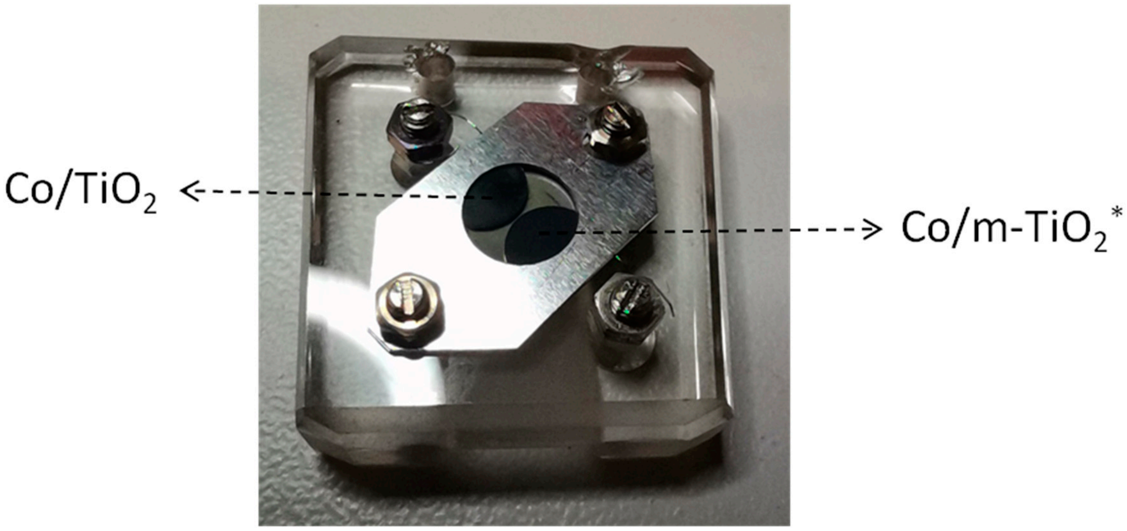

Figure S1. Photograph of the sample holder with the two catalysts mounted on it

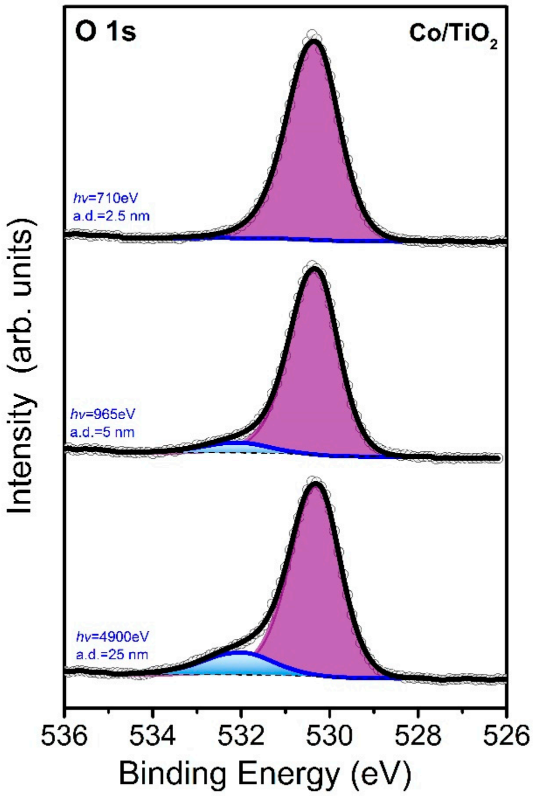

Figure S2. The O 1s spectra of Co/TiO_2 catalysts measured in 2.5 mbar CO_2:H_2 at 350 °C with 3 different excitation photon energies corresponding to 3 different analysis depths (a.d) (indicated in blue). Spectra are normalized to the same height to facilitate peak shape comparison.

Relation between photon energy and analysis depth in photoemission measurements.

The surface sensitivity of X-ray photoelectron spectroscopy is caused by the attenuation of low-energy photoelectrons due to inelastic scattering as they leave the solid sample. This attenuation is determined by the inelastic mean free path (IMFP or λ), which is the average distance an electron may travel through a material without losing energy due to inelastic scattering. In a first approximation, the Beer-Lambert

law describes the intensity I_d of a photoelectron signal (i.e. the XPS peak area) coming from an analysis depth d:

$$I_d=I_{d=0} \exp(-d/\lambda \cos\theta) \quad (1)$$

where $I_{d=0}$ is the intensity from a depth d=0, θ is the angle between the surface normal and the analyser direction (in our experiment θ=0, so cosθ=1), and λ is the IMFP. According to equation (1), 95% of electrons come from a depth within 3λ of the surface, therefore typically the analysis depth is approximated as 3λ.

The IMFP is only weakly material dependent, but rather strongly dependent on the photoelectron kinetic energy. The most common approach for calculating the IMFP as a function of electron kinetic energy (E_k) is the TPP-2M formula [1]. To better understand the correlation between E_k and λ one can use the simplified aproximative formula given by Seah and Dench [2] :

$$\lambda=c\, E_k^x \quad (2)$$

where c is a constant and x usually varies between 0.4 and 0.9 depending of the material.

For a specific core level with electron binding energy E_B, the dependence of the emitted photoelectron E_K with the incident X-ray energy (photon) as is given by Einstein's photoelectric law

$$E_K=h\nu-E_B \quad (3)$$

here hv is the photon energy, while for simplicity the contribution of the work function was omitted in equation (3).

By combining equations (2) and (3) we find:

$$\lambda=c\,(h\nu-E_B)^x \quad (4)$$

since the E_B is characteristic of the atomic core levels and independent of the excitation photon energy, is evident from equation (4) that by increasing the photon energy, λ values and consequently the analysis depth d are also increased.

References

1. Tanuma, S.; Powell, C.J.; Penn, D.R. Calculations of Electron Inelastic Mean Free Paths. V. Data for 14 Organic Compounds over the 50–2000 EV Range. *Surf. Interface Anal.* **1994**, *21*, 165–176, doi:10.1002/SIA.740210302.
2. D. Briggs (Editor), M.P.S. (Editor) *Practical Surface Analysis; Volume 1, Auger and X-Ray Photoelectron Spectroscopy*; Wiley, 1996; Vol. 1; ISBN 0471953407.
